# CD40L and IL-4 lymph node-associated signals protect B cells from rituximab-induced ADCC via KIR and NKG2A

**DOI:** 10.1093/cei/uxag001

**Published:** 2026-01-13

**Authors:** Lara V Graham, Russell B Foxall, Margaret Ashton-Key, Salim I Khakoo, Souraya Sayegh, Maria Leandro, Venkat R Reddy, Mark S Cragg, Matthew D Blunt

**Affiliations:** School of Clinical and Experimental Sciences, University of Southampton, Southampton, UK; School of Cancer Sciences, University of Southampton, Southampton, UK; Antibody and Vaccine Group, Centre for Cancer Immunology, Faculty of Medicine, University of Southampton, Southampton, UK; School of Cancer Sciences, University of Southampton, Southampton, UK; Antibody and Vaccine Group, Centre for Cancer Immunology, Faculty of Medicine, University of Southampton, Southampton, UK; School of Clinical and Experimental Sciences, University of Southampton, Southampton, UK; Deparment of Ageing, Rheumatology and Regenerative Medicine, UCL, London, UK; Department of Rheumatology, University College London Hospital, London, UK; Deparment of Ageing, Rheumatology and Regenerative Medicine, UCL, London, UK; Department of Rheumatology, University College London Hospital, London, UK; Deparment of Ageing, Rheumatology and Regenerative Medicine, UCL, London, UK; Department of Rheumatology, University College London Hospital, London, UK; School of Cancer Sciences, University of Southampton, Southampton, UK; Antibody and Vaccine Group, Centre for Cancer Immunology, Faculty of Medicine, University of Southampton, Southampton, UK; School of Clinical and Experimental Sciences, University of Southampton, Southampton, UK

**Keywords:** natural killer cells, autoimmunity, antibodies

## Abstract

Autoreactive B cells that remain in lymphatic tissue after anti-CD20 antibody therapy are considered a major contributing factor to relapse in patients with autoimmune diseases. Natural killer (NK) cells contribute to the depletion of autoreactive B cells by anti-CD20 antibodies via antibody-dependent cellular cytotoxicity (ADCC). However, the impact of germinal centre–associated signals CD40 ligand (CD40L) and interleukin-4 (IL-4) on ADCC was unknown. This study used a combination of flow cytometry, immunohistochemistry, and *ex vivo* functional assays using peripheral blood mononuclear cells to investigate how CD40L and IL-4 affect NK cell–B cell interactions. CD40L and IL-4 significantly upregulate human leukocyte antigen (HLA)-E and total HLA Class I expression on the surface of B cells from healthy donors, as well as patients with rheumatoid arthritis and systemic lupus erythematosus. The upregulation of HLA-E and total HLA functions to inhibit B-cell depletion by NK cell–mediated ADCC induced by rituximab via NKG2A and killer cell immunoglobulin-like receptors (KIR). Moreover, B cells that have differentiated through the germinal centre have higher expression of HLA-E and total HLA compared with naive B cells and are more resistant to depletion by rituximab. In accordance with this, blockade of NKG2A and inhibitory KIRs by monalizumab and lirilumab, respectively, increased antibody-dependent cellular cytotoxicity against autologous B cells *in vitro*. Overall, this study identifies a novel mechanism of resistance of B cells to NK cell cytotoxicity and indicates that blockade of the HLA-E:NKG2A and HLA:KIR checkpoint axes could be beneficial for improving B-cell depletion in patients with autoimmune diseases.

## Teaser

Autoreactive B cells that remain in lymphatic tissue after anti-CD20 antibody therapy are considered a major contributing factor to relapse in patients with autoimmune diseases. Natural killer (NK) cells contribute to the depletion of autoreactive B cells by rituximab in patients with autoimmune diseases. However, the impact of germinal centre-associated signals CD40L and IL-4 on ADCC was unknown. This study used a combination of flow cytometry, immunohistochemistry and ex vivo functional assays using PBMCs to investigate how CD40L and IL-4 affect NK cell-B cell interactions. Overall, this study identifies a novel mechanism of resistance of B cells to NK cell cytotoxicity and indicates that blockade of the HLA-E:NKG2A and HLA:KIR checkpoint axes could be beneficial for improving B cell depletion in patients with autoimmune diseases.

## Introduction

B cells have a critical role in protection against infection and cancer through antibody production, regulation of T-cell responses, and immunological memory. In the lymph nodes, naive B cells encounter antigens presented by antigen-presenting cells, leading to activation and expansion of antigen-specific B cells as part of the adaptive immune response.

On encountering antigen, primary B-cell follicles morph into secondary follicles with a germinal centre and a mantle zone. The interaction of CD40L expressed on T follicular helper (T_FH_) cells with the CD40 receptor on the B cells is a major driver of B-cell survival via upregulation of Bcl-2 family proteins [1], proliferation and formation of germinal centres [2]. Positive selection and proliferation of antigen-specific B cells through the CD40L:CD40 axis can promote somatic hypermutation of selected B cells to generate higher-affinity antibodies [3]. Interleukin-4 (IL-4) is also secreted by T_FH_ cells in the lymph nodes *in vivo* and can regulate B-cell proliferation and survival through STAT6 signalling [4, 5].

T_FH_ cells have been found to play a role in the pathogenesis of B cell–associated autoimmune diseases such as systemic lupus erythematosus (SLE) and rheumatoid arthritis (RA) [6]. These diseases are characterized by the production of high-affinity autoantibodies and are treated with B cell–depleting monoclonal antibodies (mAbs), such as the anti-CD20 mAb rituximab [7]. Anti-CD20 mAbs are effective at depleting B cells in the peripheral blood; yet residual autoimmune B cells have been demonstrated to persist in the lymphatic tissue after anti-CD20 therapy, and this is associated with poor outcomes in patients [7–9]. However, the mechanism behind this remains to be fully elucidated.

Natural killer (NK) cells are cytotoxic lymphocytes of the innate immune system that function to modulate the immune system via cytokine release and can directly lyse virus-infected cells and cancer cells. Inhibitory receptors that diminish NK cell function include NKG2A that binds HLA-E and the killer cell immunoglobulin-like receptors (KIRs) that bind classical HLA (HLA-A, B, C) expressed on target cells. NK cells are considered the predominant inducers of antibody-dependent cellular cytotoxicity (ADCC) in humans and contribute to the B cell–depleting effect of rituximab in SLE and RA patients [10]. In addition, NK cells have been detected in the lymph nodes of both humans and rodents [11], and NK cells have been shown to suppress germinal centre B cells and T_FH_ cell numbers in mice in the context of viral infection [12, 13] and vaccines [14]; although the effect of T helper cell–derived signals CD40L and IL-4 on B cell–NK cell interactions within the lymph node niche remains unknown. Here, we find that CD40L and IL-4 increase expression of HLA-E and total HLA on the surface of B cells from healthy donors, as well as from RA and SLE patients. This acts to suppress rituximab-induced B-cell depletion and NK cell–mediated ADCC via the HLA-E:NKG2A and HLA:KIR axes. In addition, memory B cells were found to have higher HLA-E and total HLA expression compared with naive B cells. Together, these data provide a novel mechanism of resistance of B cells to optimize NK cell–mediated effector functions and deplete pathogenic B cells in patients with autoimmune diseases.

## Materials and methods

### Healthy donor and patient samples

Peripheral blood mononuclear cells (PBMCs) were obtained from healthy volunteers with ethical approval from the National Research Ethics Committee (REC reference 19/WM/0262 or 24/PR/0245) after written informed consent. PBMCs from RA and SLE patients were obtained under REC reference 11/LO/1610. Patient characteristics are summarized in Table 1.

**Table 1:** SLE and RA patient characteristics.

Patient no.	Age (years)	Sex	Disease activity at time of sample collection	Previous treatments	Current treatments
SLE					
1	38	F	Active (moderate)	Hydroxychloroquine, prednisolone	Mycophenolate mofetil, prednisolone 15 mg daily, hydroxychloroquine
2	51	F	Remission	Azathioprine, NSAIDs	Hydroxychloroquine, prednisolone 5 mg daily
3	29	F	Active (severe)	Prednisolone, hydroxychloroquine	Mycophenolate mofetil, prednisolone 30 mg daily, azathioprine
4	44	F	Low activity	Hydroxychloroquine	Hydroxychloroquine, prednisolone 7.5 mg daily
5	35	F	Active (moderate)	Azathioprine, prednisolone	Mycophenolate mofetil, prednisolone 20 mg daily, hydroxychloroquine
6	47	F	Remission	NSAIDs, prednisolone	Hydroxychloroquine, prednisolone 5 mg daily
RA					
1	62	F	Active (moderate)	Methotrexate, NSAIDs	Methotrexate 15 mg weekly, sulfasalazine, prednisolone 7.5 mg daily
2	55	M	Low activity	Methotrexate	Methotrexate 20 mg weekly, prednisolone 5 mg daily
3	48	F	Active (severe)	Methotrexate, NSAIDs	Methotrexate 15 mg weekly, azathioprine, prednisolone 10 mg daily
4	71	F	Remission	Leflunomide, NSAIDs	Leflunomide 20 mg daily, prednisolone 5 mg daily
5	52	F	Active (moderate)	Methotrexate, prednisolone	Methotrexate 15 mg weekly, sulfasalazine, prednisolone 7.5 mg daily
6	59	M	Low activity	Methotrexate, NSAIDs	Methotrexate 20 mg weekly, hydroxychloroquine, prednisolone 5 mg daily

F, female; M, male; NSAIDs, non-steroidal anti-inflammatory drugs.

### Flow cytometry

Healthy donor PBMCs were incubated with 300 ng/ml CD40L and 10 ng/ml IL-4 (both R&D systems) for 24 h at 37°C 5% CO_2_, as previously described [15]. PBMCs were washed twice and stained with fluorescent antibodies (Table 2) in fluorescence activated cell sorting (FACS) buffer (phosphate buffered saline, bovine serum albumin 1%, sodium azide 0.05%) at 4°C for 30 min. Data were collected on a BD FACS Aria II (BD Biosciences) using FACSDiva software (BD Biosciences) and analysed with FlowJo v10.8.1 (BD Bosciences).

**Table 2:** Fluorescent antibodies to analyse human cells by flow cytometry.

Target	Fluorochrome	Isotype	Clone	Supplier	Concentration (μg/ml)
HLA-E	PE/Cy7	mIgG1	3D12	BioLegend	6.00
CD107a	eFluor660	mIgG1	eBioH4A3	eBioscience	0.17
CD14	PE	mIgG2a	M5E2	BioLegend	4.00
CD19	Pacific blue	mIgG1	HIB19	BioLegend	2.00
CD27	PE	mIgG1	M-T271	BioLegend	2.00
CD3	PerCP	mIgG1	UCHT1	BioLegend	1.00
CD56	PE/Cy7 or PE	mIgG1	HCD56	BioLegend	4.00
IgG	APC	rIgG2a	M1310GU5	BioLegend	0.50
KIR2DL3/L2/S2 (CD158b)	PE	mIgG2b	CH-L	BD Biosciences	1.30
KIR2DL1/S1 (CD158a)	PE	mIgG1	11PB6	Miltenyi Biotech	4.00
NKG2A	FITC/Vio Bright V423	hIgG1	REA110	Miltenyi Biotech	1.00
Total HLA	AF488	mIgG2a	W6/32	BioLegend	0.30

### NK-cell degranulation

PBMCs from healthy human donors were treated with CD40L and IL-4 as above. PBMC samples were stained with 0.17 μg/ml α-CD107a-eFluor660 (eBioH4A3, Invitrogen) for 10 min at room temperature and incubated with 10 μg/ml rituximab or isotype control (AT171–2, both in-house) for 24 h at 37°C with addition of Golgistop (BD Biosciences) after 1 h. In indicated experiments, 10 μg/ml Z199 (Beckman Coulter), monalizumab (Stratech), or lirilumab (BMS) was added 20 min before the addition of rituximab. Data were collected using flow cytometry as above.

### B-cell depletion in human PBMCs

Healthy donor PBMCs were incubated with the indicated concentrations of rituximab for 24 h at 37°C 5% CO_2_ and B-cell populations that remained were assessed by flow cytometry or healthy donor PBMCs were incubated with CD40L and IL-4 as above for 24 h before the addition of rituximab or isotype control for a further 24 h at 37°C 5% CO_2_. The proportion of B cells (CD3-CD19^+^) and T cells (CD3^+^CD19^−^) remaining after 24 h was identified by flow cytometry as above. B-cell depletion was evaluated using samples not treated with antibody as described previously [10] as follows:


$$\begin{array}{rl} & B\text{-}\mathrm{cell}\;\mathrm{depletion}=100-[\left(\frac{100}{B:T\;\mathrm{cell}\;\mathrm{ratio}\;\mathrm{in}\;\mathrm{control}}\right)\\ & \;\times (B:T\;\mathrm{cell}\;\mathrm{ratio}\;\mathrm{in}\;\mathrm{sample}\;\mathrm{containing}\;\mathrm{antibody})] \end{array}$$

### Immunohistochemistry

Formalin-fixed, paraffin-embedded (FFPE) sections (4 μm) of a consented patient lymph node pre-treatment were provided by Cellular Pathology, University Hospital Southampton NHS Foundation Trust. Ethical approval was obtained by Southampton University Hospitals NHS Trust from Southampton and Southwest Hampshire Research Ethics Committee (REC reference 23/NW/0060). Sections were stained using the fully automated BOND-RX immunohistochemistry staining instrument (Leica Microsystems), in accordance with the manufacturer’s instructions. In brief, sections were deparaffinized, pre-treated for heat-induced antigen retrieval and stained for HLA-E (MEM-E/02, Abcam) or CD20 (L26, DAKO) in BOND Primary Antibody Diluent (Leica Microsystems). The antibody was then bound to the polyHRP IgG reagent before incubation with 3,3′-diaminobenzidine. Slides were digitalized using the Zeiss Axioscan 7 and images were taken using Zenv3.10 Lite (Zeiss).

### Statistics

Statistical analysis was performed using GraphPad Prism software (version 10.0). One-way or two-way analysis of variance (ANOVA) was used to compare data with more than two groups. Data were considered statistically significant at *P* < 0.05.

## Results

### CD40L and IL-4 upregulate HLA-E and total HLA expression on the surface of B cells from healthy donors

**Figure 1:**
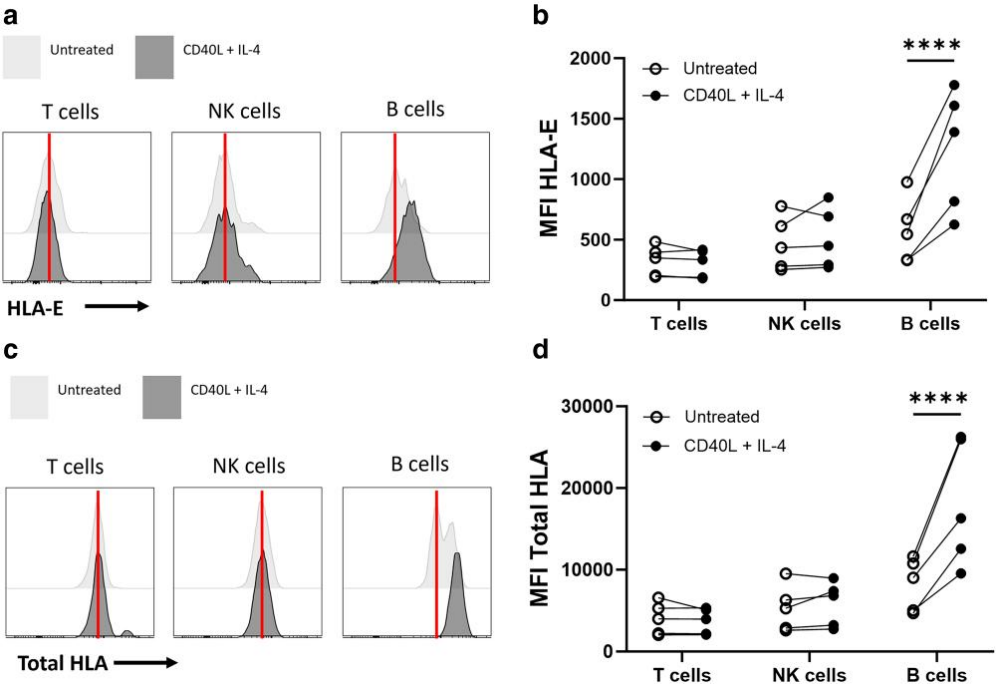
CD40L and IL-4 lymph node support signals increase HLA-E and pan-HLA expression on the surface of healthy donor B cells. (a–d) PBMCs from healthy human donors were incubated with 300 ng/ml CD40L and 10 ng/ml IL-4 for 24 h. HLA-E and total HLA expression on T cells, NK cells, B cells, and monocytes was assessed by flow cytometry. Representative FACS plots are shown in (a) and (c), and MFI data are summarized in (b) and (d) (*n* = 5). Analysed by two-way ANOVA (with Sidak’s correction for multiple comparisons). ****P < 0.0001.

We recently identified that CD40L and IL-4 upregulate HLA-E on the surface of primary chronic lymphocytic leukaemia cells and non-Hodgkin’s lymphoma cell lines [16, 17]. Therefore, we first asked whether the effect of CD40L and IL-4 on HLA-E expression is also seen in healthy B cells. PBMCs from healthy human donors were treated with CD40L and IL-4 for 24 h and expression of HLA-E, and total HLA was assessed by flow cytometry. CD40L and IL-4 had no effect on expression of HLA-E on the surface of T cells and NK cells (Fig. 1a and b), as expected as these cells lack expression of CD40 [3]. Similar to previous observations with malignant B cells [16, 17], HLA-E increased, on average, over 2-fold on the surface of healthy B cells (*P* < 0.0001). Comparable results were shown for total HLA (Fig. 1c and d). This indicates that CD40L and IL-4 can alter the expression of ligands for NK cells on B cells.

### HLA-E and total HLA surface expression are increased on post-germinal centre B cells in healthy donors

**Figure 2:**
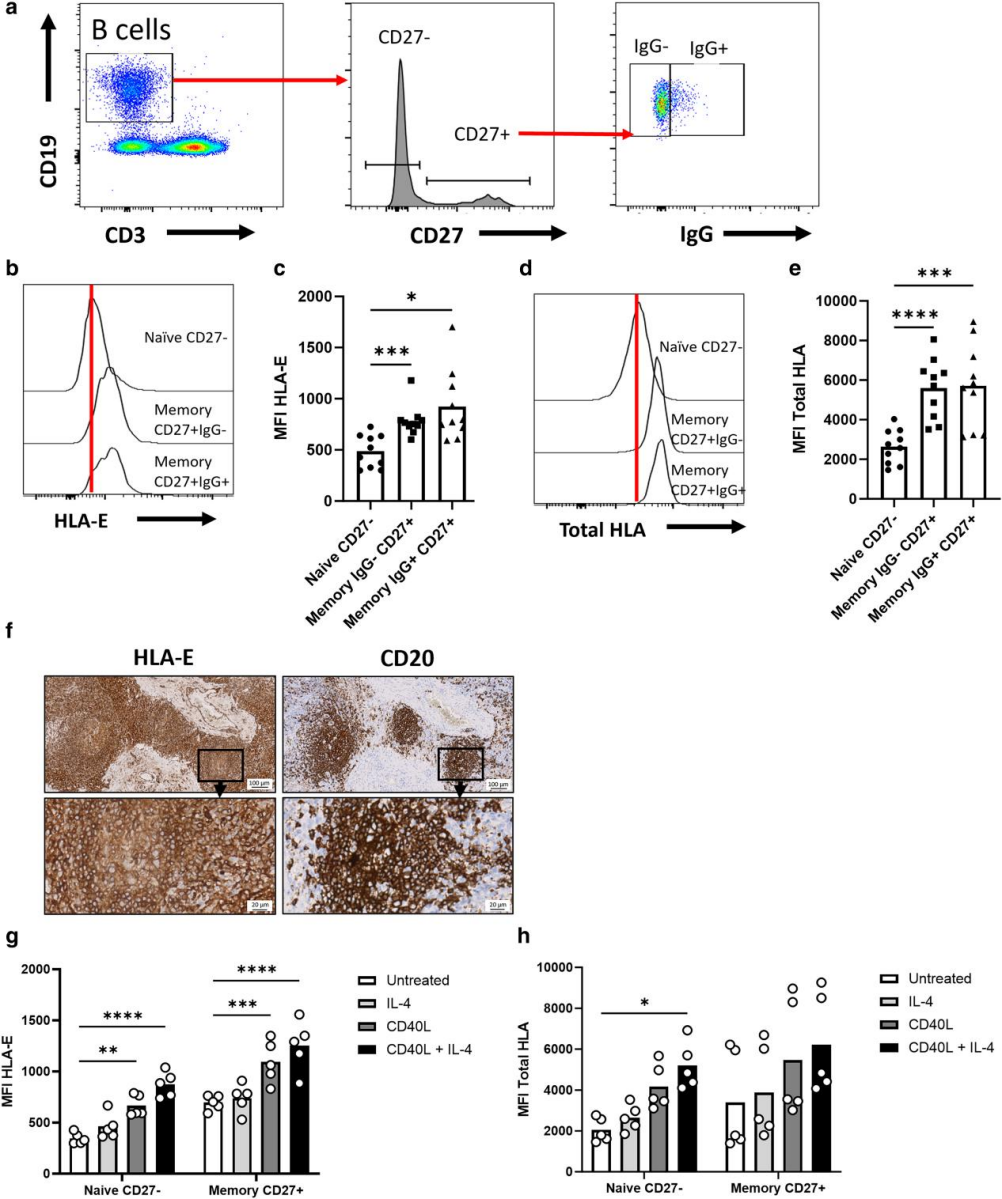
Post-germinal centre CD27 + memory B cells display higher HLA-E expression than CD27^−^ naive B cells. (a–e) HLA-E and total HLA expression were assessed on untreated naive (CD27^−^), unswitched (CD27^+^IgG^−^) or isotype-switched (CD27^+^IgG^+^) memory B cells by flow cytometry. Gating strategy shown in (a). Representative HLA-E and total HLA data are shown in (b) and (d), and MFI data are summarized in (c) and (e) (*n* = 10). (f) FFPE lymph node sections from a consented patient without a B-cell malignancy diagnosis were stained with antibodies for human HLA-E and CD20. Images taken at ×4 magnification (top panel, scale bar 100 μm) and image of selected secondary follicle taken at ×16 magnification (bottom panel, scale bar 20 μm). (g and h) PBMCs from healthy human donors were incubated with 10 ng/ml IL-4, 300 ng/ml CD40L, CD40L, and IL-4 combined or left untreated for 24 h. HLA-E (g) and total HLA (h) expression on naive (CD27^−^) and memory (CD27^+^) B cells was assessed by flow cytometry. Data are summarized as mean ± SEM in (g) and (h) (*n* = 5). Analysed by one-way or two-way ANOVA (with Tukey’s or Dunnet’s correction for multiple comparisons). *P < 0.05, **P < 0.01, ***P < 0.001, ****P < 0.0001.

To assess whether B-cell maturation *in vivo* affects the expression of HLA-E and total HLA, we next examined expression of these ligands on untreated naive (CD27^−^) B cells, memory unswitched (CD27^+^IgG^−^) and memory isotype-switched (CD27^+^IgG^+^) B cells (Fig. 2a). Switched and unswitched memory B cells had significantly higher expression of both HLA-E (mean MFI 922 for switched and 784 for unswitched, *P* < 0.05 and *P* < 0.001) and total HLA (mean MFI 5720 for switched and 5590 for unswitched, *P* < 0.001 and *P* < 0.0001) compared with naive B cells (mean MFI 486 for HLA-E and 2639 for total HLA, Fig. 2b–e). This indicates that there is an increase in expression of HLA-E and total HLA on the surface of post-germinal centre B cells in humans. Expression of HLA-E was also confirmed on CD20^+^ B cells, as well as CD20^−^ cells, in secondary lymphoid follicles in a lymph node biopsy negative for cancer (Fig. 2f). This is in accordance with previous reports [18] and correlates with our PBMC data (Fig. 1a and b). Both naive (CD27^−^) and memory (CD27^+^) B cells responded similarly to stimulation with CD40L and IL-4. HLA-E (*P* < 0.001 and *P* < 0.0001) and total HLA (*P* < 0.01 and *P* < 0.05) significantly increased in response to CD40L and IL-4 combined (Fig. 2g and h). CD40L treatment alone was also able to significantly increase expression of HLA-E (*P* < 0.05 and *P* < 0.01) and total HLA (naive *P* < 0.05), whereas there was no significant increase with IL-4 alone (Fig. 2g and h).

### CD40L and IL-4 upregulate HLA-E and total HLA expression on the surface of B cells from RA and SLE patients

**Figure 3:**
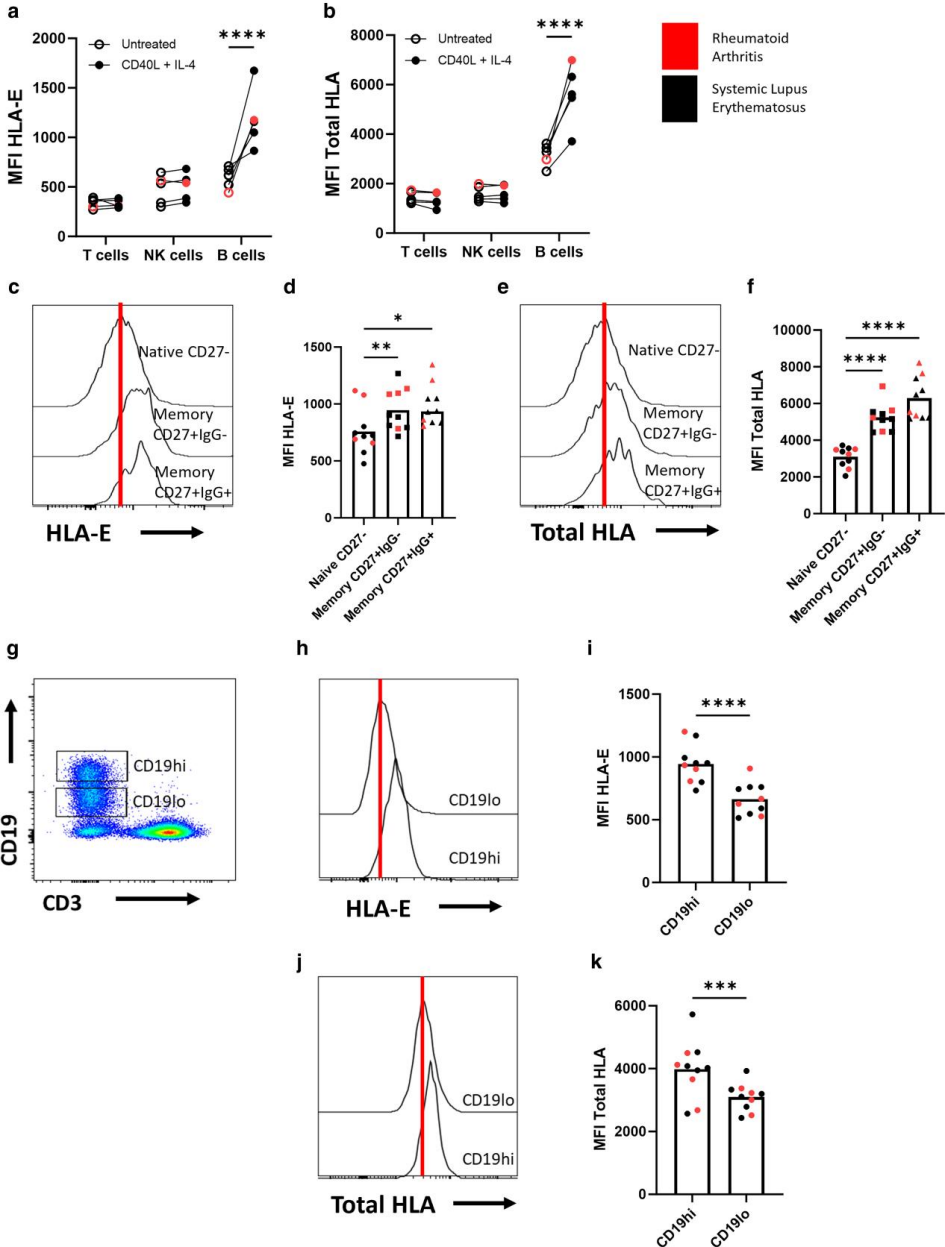
CD40L and IL-4 increases HLA-E and total HLA expression on B cells from RA and SLE patients. (a and b) PBMCs from RA and SLE patients were incubated with 300 ng/ml CD40L and 10 ng/ml IL-4 for 24 h. HLA-E (a) and total HLA (b) expression on T cells, NK cells, and B cells from RA and SLE patients were assessed by flow cytometry (*n* = 5). (c–f) HLA-E (c, d) and total HLA (e, f) expression were assessed on naive (CD27^−^), unswitched (CD27^+^IgG^−^) or isotype-switched (CD27^+^IgG^+^) memory B cells by flow cytometry (*n* = 10). (g) Gating strategy to identify B cells expressing high (CD19hi) and low (CD19lo) levels of CD19 by flow cytometry. HLA-E (h, i) and total HLA (j, k) expression were assessed on CD19hi and CD19lo B cells from RA and SLE patients by flow cytometry (*n* = 10). Analysed by paired *t* test or one-way ANOVA (with Dunnet’s correction for multiple comparisons). *P < 0.05, **P < 0.01, ***P < 0.001, ****P < 0.0001.

Anti-CD20 antibodies, including rituximab, are extensively used for the treatment of B cell–related autoimmune conditions such as SLE and RA. Moreover, incomplete depletion of autoreactive B cells by anti-CD20 antibodies in the lymph node compartment is associated with poorer outcomes in these diseases [7, 8]. However, the mechanism behind this is unknown. It was therefore important to assess whether the effect of CD40L and IL-4 lymph node–associated signals on HLA expression in healthy B cells was reflected in B cells from RA and SLE patients. In agreement with healthy donor PBMCs (Fig. 1), CD40L and IL-4 significantly increased HLA-E (mean MFI 593 rising to 1184, Fig. 3a) and total HLA (mean MFI 3162 rising to 5620, Fig. 3b) expression on the surface of B cells from RA and SLE patients (*P* < 0.0001). HLA-E expression was also significantly higher on memory CD27^+^IgG^−^ (*P* < 0.01) and IgG^+^ (*P* < 0.05) B cells (Fig. 3c and d), as well as total HLA (*P* < 0.0001, Fig. 3e and f) compared with CD27^−^ naive B cells. In addition, B cells expressing high levels of CD19 (CD19^hi^; Fig. 3g) have been identified as having a higher potential to promote inflammation in autoimmune diseases [19] and are thus considered more pathogenic than B cells with lower CD19 expression (CD19^lo^). CD19^hi^ B cells from RA and SLE patients also had significantly higher surface expression of HLA-E (mean MFI 945, *P* < 0.0001 Fig. 3h and i) and total HLA (mean MFI 3983, *P* < 0.001, Fig. 3j and k) compared with CD19^lo^ B cells (mean MFI 665 for HLA-E and 3091 for total HLA). These data demonstrate that T helper cell–derived signals can upregulate HLA-E and total HLA on the surface of B cells from healthy donors and patients with autoimmune diseases.

### CD40L and IL-4 lymph node–associated signals protect from rituximab-induced autologous B-cell depletion and suppress NK cell–mediated ADCC

**Figure 4:**
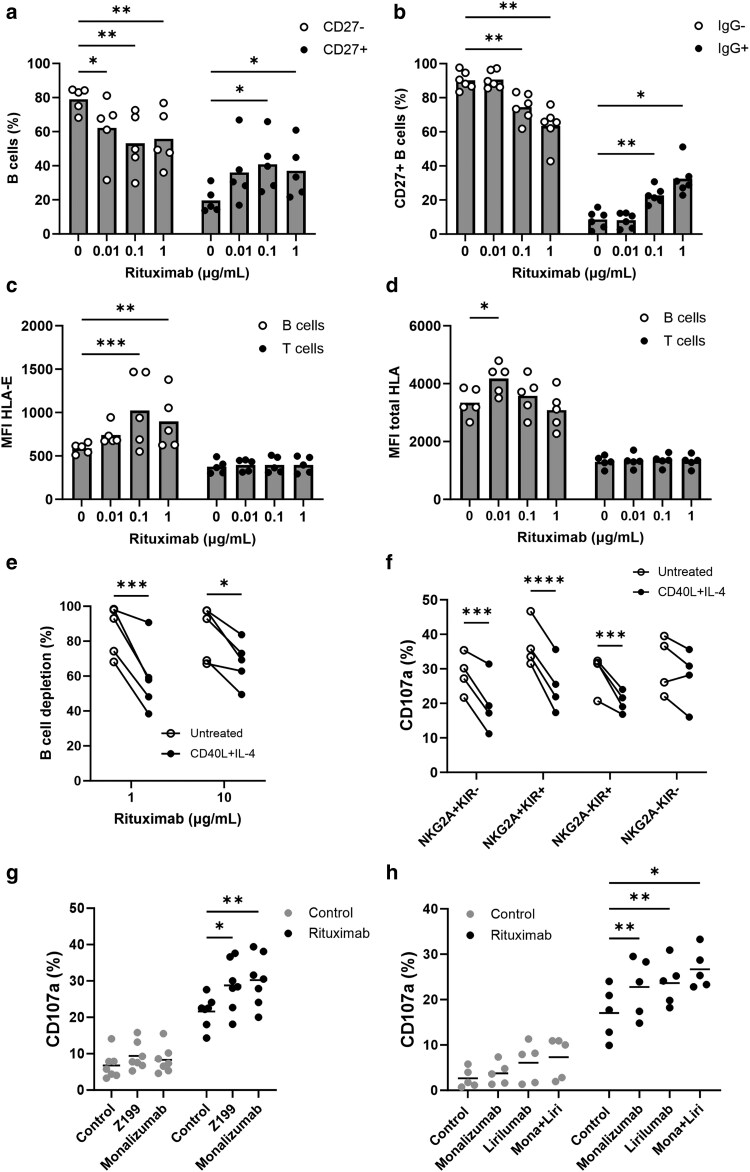
CD40L and IL-4 treatment inhibits rituximab-mediated B-cell depletion and NK cell–mediated ADCC via the HLA-E:NKG2A and HLA:KIR axes. (a–d) Healthy human PBMCs were treated with the indicated concentrations of rituximab for 24 h and the percentage of (a) CD27^−^/CD27^+^ or (b) CD27^+^IgG^−^/IgG^+^ were measured by flow cytometry (*n* = 5). Flow cytometry was also used to measure (c) HLA-E and (d) total HLA expression on the surface of B and T cells post-rituximab treatment (*n* = 5). (e) Healthy human PBMCs were treated with CD40L and IL-4 for 24 h before addition of rituximab (1 or 10 μg/ml) or isotype control for a further 24 h. The proportion of B cells to T cells post-rituximab treatment was measured by flow cytometry (*n* = 5). (f) Healthy human PBMCs were treated with CD40L and IL-4 for 24 h or left untreated before addition of 10 μg/ml rituximab for a further 4 h. Degranulation (CD107a expression) of NKG2A^+^KIR^−^, NKG2A^+^KIR^+^, NKG2A^−^KIR^+^, and NKG2A^−^-KIR^−^ NK cells was measured by flow cytometry (*n* = 4). (g) Healthy human PBMCs were treated with CD40L and IL-4 for 24 h before treatment with 10 μg/ml rituximab or isotype control in combination with 10 μg/ml anti-NKG2A antibodies monalizumab or Z199. (h) Healthy human PBMCs were treated with CD40L and IL-4 for 24 h before treatment with 10 μg/ml rituximab or isotype control in combination with 10 μg/ml monalizumab, lirilumab, or both. Statistical significance was calculated using two-way ANOVA (with Dunnet’s or Sidak’s correction for multiple comparisons). *P < 0.05, **P < 0.01, ***P < 0.001, ****P < 0.0001.

HLA-E and total HLA are ligands for the inhibitory receptors NKG2A and KIRs expressed on NK cells. To assess whether levels of HLA-E and total HLA could affect NK cell cytotoxicity against autologous B cells, we examined the relative proportions of surviving B cells in an autologous B-cell depletion assay with rituximab. As untreated CD27^+^ and IgG^+^ memory B cells displayed higher levels of HLA-E and total HLA, we hypothesized that these cells would be less well depleted by rituximab. We found that the proportion of both CD27^+^ (Fig. 4a) and IgG^+^ (Fig. 4b) B cells significantly increased with increasing concentration of rituximab, indicating these cells are less well depleted by rituximab. Interestingly, rituximab enriched for B cells expressing higher levels of HLA-E (*P* < 0.001 for 0.1 μg/ml or *P* < 0.01 for 1 μg/ml; Fig. 4c) and, to a lesser extent, total HLA (*P* < 0.05 for 0.01 μg/ml; Fig. 4d). HLA-E and total HLA expression remained stable on T cells, in accordance with these cells not being the target of rituximab (Fig. 4c and d). These data indicate that B cells with higher expression of HLA-E and total HLA are more resistant to NK cell cytotoxicity and are better able to survive rituximab treatment.

As CD40L and IL-4 increased HLA-E and total HLA on the surface of healthy human B cells, we next tested whether CD40L and IL-4 treatment could affect B-cell depletion induced by rituximab. Healthy human PBMCs were treated with CD40L and IL-4 for 24 h before subsequent culture with either rituximab (1 or 10 μg/ml) or isotype control, and rituximab-dependent B-cell depletion was assessed using flow cytometry. Treatment of PBMCs with CD40L and IL-4 significantly reduced B-cell depletion in the presence of 1 μg/ml (mean depletion 86% reducing to 59%, *P* < 0.001) or 10 μg/ml (mean depletion 85% reducing to 67%, *P* < 0.05) rituximab (Fig. 4e), indicating T helper cell–derived signals can induce resistance to rituximab-induced B-cell depletion.

### Blockade of NKG2A and/or KIRs can potentiate NK cell–mediated ADCC against autologous B cells

As NK cells are a predominant mediator of rituximab function in humans, we hypothesized that upregulation of HLA-E and total HLA by CD40L and IL-4 inhibits NK cell–mediated ADCC of autologous B cells via the HLA-E:NKG2A and HLA:KIR axes. To test whether CD40L and IL-4 treatment could affect ADCC by NK cells, we measured degranulation (CD107a) by the NK cells in combination with rituximab. CD40L and IL-4 treatment significantly decreased degranulation of NKG2A^+^KIR^−^ (*P* < 0.001), NKG2A^+^KIR^+^ (*P* < 0.0001) and NKG2A^−^KIR^+^ (*P* < 0.001) NK cells, but not NKG2A^-^KIR^-^ NK cells (Fig. 4f), indicating inhibition of NK cell–mediated ADCC by CD40L and IL-4 is via the HLA-E:NKG2A and HLA:KIR axes. As NKG2A and KIR blockade are currently under clinical evaluation in cancer (NCT05221840, NCT01592370, and NCT02399917), we tested whether NKG2A and KIR blocking mAbs could improve NK cell–mediated ADCC against autologous B cells treated with CD40L and IL-4. The anti-NKG2A mAbs Z199 (*P* < 0.05) and clinically relevant monalizumab (*P* < 0.01) were able to significantly increase NK cell degranulation in combination with rituximab (Fig. 4g). In addition, the anti-KIR mAb lirilumab was able to significantly increase NK cell–mediated ADCC (*P* < 0.01, Fig. 4h). The combination of lirilumab and monalizumab also induced significantly higher NK cell activation with rituximab compared with the control (*P* < 0.05); however, the difference between the combination and either lirilumab or monalizumab alone was not statistically significant (Fig. 4h). Finally, NK cells from RA and SLE patients expressed both NKG2A and KIRs (Supplementary Fig. S1a and b), indicating that these receptors could be novel immune checkpoint targets in these diseases. Overall, these data indicate upregulation of HLA-E and total HLA on the surface of B cells from healthy donors can inhibit autologous B-cell depletion and NK cell–mediated ADCC in combination with rituximab *in vitro*, and that this can be improved by NKG2A and/or KIR blockade.

## Discussion

NK cells are important in mediating rituximab-induced B-cell depletion in patients with RA and SLE and have been shown to suppress germinal centre B cells in mice. However, the effects of CD40L and IL-4 lymph node–associated signals on NK–B cell interactions in humans were unknown. This study finds that CD40L and IL-4 increase expression of the NK-cell inhibitory ligands HLA-E and total HLA on B cells from both healthy donor and RA and SLE patient samples and reduce rituximab-mediated B-cell depletion. Furthermore, NK-cell function against autologous B cells was inhibited by IL-4 and CD40L through the HLA-E:NKG2A and HLA:KIR axes. Both KIR and NKG2A blockade were able to improve NK cell–mediated ADCC. Taken together, these data identify a novel mechanism of resistance of B cells to NK cell cytotoxicity and demonstrate that the combination of NKG2A and/or KIR blockade with rituximab may deepen B-cell depletion in patients with autoimmune diseases.

CD40L and IL-4 from T-helper cells within the lymph nodes are important for driving germinal centre formation and B-cell maturation to generate antigen-specific plasma and memory B cells. Previous studies have identified that CD40L and IL-4 stimulation *ex vivo* can polarize B cells to an antigen-presenting phenotype [20] and induce upregulation of anti-apoptotic proteins critical for maintaining B-cell survival [1]. Our study adds to this by demonstrating upregulation of HLA molecules on the surface of B cells from healthy donors. NK cells have previously been demonstrated to suppress germinal centre B cells in mice with viral infection or receiving vaccines, thought to be primarily due to cytotoxicity against CD4^+^ T cells [12–14]. As memory B cells were found to have higher HLA-E and total HLA expression compared with naive B cells, upregulation of HLA molecules within germinal centres may represent a novel mechanism of protection for post-germinal centre B cells from NK cell cytotoxicity, although this has not been directly confirmed. Because the *in vitro* models used in this study do not fully recapitulate the lymph node microenvironment or germinal centre architecture, future work should aim to investigate this mechanism utilizing 3D lymph node–mimicking models *in vitro* and/or appropriate murine models *in vivo*.

We also demonstrate inhibition of NK cell–mediated ADCC with CD40L and IL-4 which may have implications for the efficacy of rituximab therapy in patients with B cell–related autoimmune diseases. Previous reports suggest that rituximab-induced depletion of B cells in patients with autoimmune diseases is less effective in the lymph nodes relative to peripheral blood [8]. Furthermore, isotype-switched memory B cells were more resistant to rituximab-mediated depletion than naive B cells in patients receiving kidney transplants [21]. In agreement, isotype-switched memory B cells were found to persist in the lymph nodes of RA patients after rituximab treatment [22] and higher frequency of isotype-switched memory B cells is associated with poor clinical response to rituximab in RA [10]. Our data identify a potential NK cell–associated mechanism for this via increased expression of ligands for the KIRs and NKG2A. However, this is yet to be confirmed *in vivo* and could be investigated through the analysis of lymph node biopsies from patients with autoimmune diseases.

B–T cell collaboration through the CD40L:CD40 axis in the lymph nodes and affected joints has been identified as an important pathological signal in autoimmune diseases [23]. Moreover, higher proportions of T_FH_ cells and B cells were found in the lymph nodes of RA patients and patients at risk of developing RA [24, 25], indicating changes within the lymph node may be an early marker of disease development and progression. Attempts to dampen B–T cell collaboration in autoimmune diseases have been made with the anti-CD40L antibody dapirolizumab (NCT04294667) and depletion of T_FH_ cells with an anti-PD-L1 CAR-NK [26]. Alternatively, the data in this study reveal that blockade of the inhibitory KIR and NKG2A checkpoints may aid rituximab responses in patients due to suppression of NK cell–mediated ADCC through these immune checkpoints induced by CD40L and IL-4. The anti-NKG2A antibody monalizumab is currently in clinical trials for patients with lung cancer (NCT05221840) and the results of this study indicate that the addition of monalizumab to rituximab therapy may also potentially be beneficial for improving NK cell cytotoxicity and rituximab efficacy in patients with autoimmune diseases, although the clinical relevance of this remains to be assessed. Lirilumab is also currently in an active Phase II clinical trial (NCT03341936) for squamous cell carcinoma of the head and neck. Therefore, combining monalizumab or lirilumab with rituximab in patients with RA or SLE may have clinical utility and could be tested in clinical trials seeking to enhance B-cell depletion within lymph nodes. Moreover, B cell–targeting chimeric antigen receptor (CAR)-T cells and CAR-NK cells are being evaluated for the treatment of autoimmune diseases and are both currently in clinical trials for B cell–related autoimmune diseases [27]. To generate sufficient numbers of CAR-NK cells for therapy, these must be expanded *ex vivo* [28] and this process is known to upregulate NKG2A expression [29, 30]. In addition, CAR-T cells have been shown to upregulate NKG2A post-infusion into cancer patients [31]. This indicates that interruption of the HLA-E:NKG2A checkpoint axis may have relevance for the improvement of depletion of autoreactive B cells by CAR-based cellular therapies.

## Conclusion

In conclusion, CD40L and IL-4 lymph node–associated signals upregulate expression of HLA-E and total HLA on the surface of B cells from healthy donors and patients with autoimmune diseases. This functions to inhibit rituximab-induced ADCC by NK cells via the HLA-E:NKG2A and HLA:KIR axes. Blockade of these immune checkpoints in combination with rituximab may allow for deepened B-cell depletion within the lymph node compartment of patients with autoimmune diseases. Overall, this study provides new insight into NK–B cell interactions within the lymph node microenvironment and reveals a novel mechanism of resistance for B cells to NK cell–mediated ADCC.

## Supplementary Material

uxag001_Supplementary_Data

## Data Availability

All data generated or analysed during this study are included in this published article (and its Supplementary material files) and the raw data will be made freely available upon request.
