# Supplementary material for: CD40L and IL-4 lymph node-associated signals protect B cells from rituximab-induced ADCC via KIR and NKG2A
Source: Clin Exp Immunol. 2026 Jan 13;220(1):uxag001. doi: 10.1093/cei/uxag001 (PMC12865455; doi:10.1093/cei/uxag001)
Supplement: uxag001_Supplementary_Data [file uxag001_supplementary_data.docx]

Supplementary

| **A** | **B** |
| --- | --- |
| 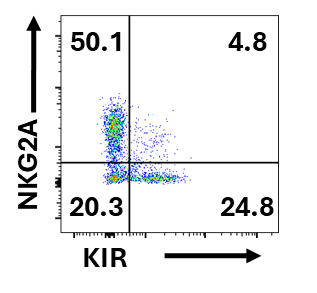 |  |
| **Figure S1: NKG2A and KIR expression on NK cells from rheumatoid arthritis and systemic lupus erythematosus patients.**  NKG2A and KIR (KIR2DL3/L2/L1/S2/S1) expression on peripheral blood CD56+ CD3-NK cells from rheumatoid arthritis (RA) and systemic lupus erythematosus (SLE) patients was measured by flow cytometry. Representative FACS plots with relative proportions shown in A and summarised data shown in B. Red dots represent samples from RA patients (n=6) and black dots represent samples from SLE patients (n=6). | |
